# Blood-Stained Colostrum: A Rare Phenomenon at an Early Lactation Stage

**DOI:** 10.3390/children9020213

**Published:** 2022-02-06

**Authors:** Wszołek Katarzyna, Pięt Małgorzata, Więckowska (Pająk) Agata, Meissner Wioletta, Mazela Jan, Rybicka Katarzyna, Wilczak Maciej

**Affiliations:** 1 Gynecological and Obstetrics Hospital, Poznan University of Medical Sciences, 61-701 Poznań, Poland; mpiet@ump.edu.pl (P.M.); wioletta.meissner@gmail.com (M.W.); janco@pol-med.com.pl (M.J.); kasia_rybicka7@wp.pl (R.K.); mwil@gpsk.ump.edu.pl (W.M.); 2 Department of Maternal and Child Health, Poznan University of Medical Sciences, 60-535 Poznań, Poland; 3 Department of Midwifery, Poznan University of Medical Sciences, 60-535 Poznań, Poland; 4 Department of Neonatology, St. Hedwig of Silesia Hospital, 55-100 Trzebnica, Poland; agata_pa@wp.pl; 5 Neonatal Infection Clinic, Gynecological and Obstetrics Hospital, Poznan University of Medical Sciences, 60-535 Poznań, Poland

**Keywords:** blood, colostrum, nipple secretion, breastfeeding, lactation, rusty pipe syndrome

## Abstract

The phenomenon described in the literature as rusty pipe syndrome is a rare condition (the estimated incidence is 0.1% in the population of breastfeeding women) where the prenatal milk and the colostrum are rust- or blood-colored. Due to the rare occurrence of this phenomenon and the related nature of the baby’s regurgitated discharge—green, brown or blood-stained, there is a general fear of latching a newborn or continuing to breastfeed if the regurgitation persists. In this care report, a patient’s milk was tested to determine its microbiological and morphological content. No significant abnormalities were noted in these tests. The nutritional profile of the blood-stained colostrum was normal. Breast milk has an indisputably invaluable impact on the newborn’s further development and there is no connection between rusty pipe syndrome, as described in the literature, and any clinical complications. This is crucial to encourage mothers to keep breastfeeding even if they observe blood-stained colostrum.

## 1. Background

Depending on the stage of lactation, milk can be identified as prenatal milk, colostrum, transitional milk, and mature milk [1,2]. Prenatal milk is secreted during lactogenesis I, and its emergence results from the penetration of the intercellular space by lactocyte secretion [3]. Colostrum is secreted starting from days 0 to 5 after childbirth and is a thick, usually yellowish liquid [4]. 

The phenomenon described in the literature as rusty pipe syndrome is a rare condition where the prenatal milk and the colostrum are rust- or blood-colored and the presence of erythrocytes in the discharge of one or both breasts is not connected with any mechanical damage of the nipple or with the presence of intraductal papilloma [5,6,7]. In this condition, the mother does not feel any pain and the symptoms partially disappear spontaneously 5–10 days after childbirth [8,9,10]. Breastfeeding is fully safe and recommended if the newborn tolerates the milk and shows no signs of irritation of the gastrointestinal tract and the pediatrician has no reasons to suspect any causes of brownish regurgitation liquid other than the consumption of erythrocyte-containing colostrum [6,10,11,12]. 

## 2. Case Presentation

A 40-week-pregnant 29-year-old primipara was referred to hospital by the attending physician, with the diagnosis: 1st pregnancy (Grav 1), 40 pregnancy weeks (40 Hbd), type A1 gestational diabetes (diet modification was sufficient, patient did not need the additional insulin injections), hearing impairment. Spontaneous vaginal birth took place in the 41st week of pregnancy. A female neonate was born vaginally with a birth weight of 3180 g. Clean amniotic fluid was released an hour before labor, while the hindwaters were green. The newborn was given Apgar scores of 8/8/10/10 after 1/3/5/10 min of life, respectively. In order to rule out meconium aspiration syndrome, the newborn was transferred to the Intermediate Care Unit for observation. The infant received milk formula for the first feeding before it was reunited with the mother in the rooming-in unit. 

**Figure 1:**
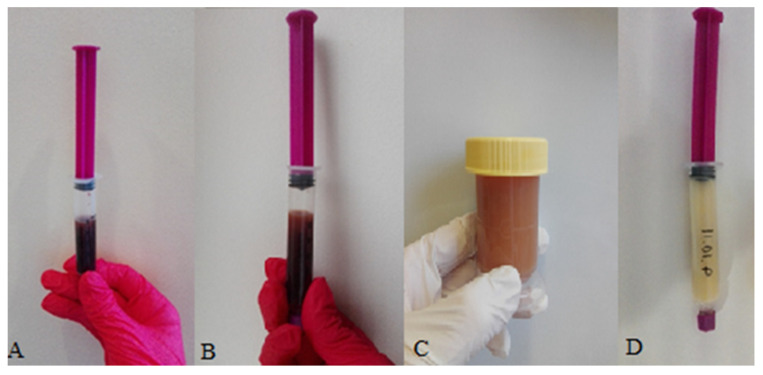
The patient’s colostrum color changes in the following lactation days: (**A**) 1st day, (**B**) 2nd day, (**C**) 3rd day, and (**D**) 4th day.

The mother observed a brown-colored nipple secretion from both breasts (Figure 1).

Since the brownish breast discharge persisted, a lactation consultant was asked to give their opinion. After taking the medical history and identifying the blood-stained colostrum from both the right and left breasts, both the consulting midwife and neonatologist ordered breastfeeding to continue, as there were no contraindications to natural feeding. 

Due to the patient’s positive medical history of benign breast lesions (fibroadenoma—confirmed in left breast biopsy) and tendency towards bruising and recurring gingival bleeding, the colostrum was sampled from both breasts for a cytological test. The cytological test revealed amorphous acidophilous content, mononuclear macrophages and erythrocytes, and no atypical cells in both breasts. Additionally, microbiological and blood tests were performed. The microbiological test identified *Staphylococcus hominis* and *Streptococcus salivarius* in the milk samples from the right and left breasts. Complete blood tests revealed low platelet counts both before and after childbirth. The platelet amount before childbirth was 158.0 G/L and two days later it was 138.0 G/L, whereas a normal platelet count is 150.0–400.0 G/L.

On the 2nd day after childbirth, lactogenesis II progressed normally. The colostrum still had a bloody brown color (Figure 1). The newborn’s body weight loss was normal (170 g from the birth weight) and the child passed urine and stool. 

On the 3rd day from birth, breast fullness was observed and the baby gained 10 g compared to the previous day (−160 g from the birth weight). The newborn no longer regurgitated and it passed urine and meconium properly (still dark). The child’s general parameters were normal. Physical examination revealed a good general condition; a non-distended, soft abdomen; and mild jaundice. On the 4th day of life, the baby was discharged home in a stable condition. 

On the third day following birth, the color of the colostrum changed to rusty (Figure 1). The mother reported that the colostrum color had become normal—i.e., yellow—on the 5th day following birth.

The composition of the patient’s milk was analyzed using the Miris HMA (Miris AB, Sweden) on the 2nd, 3rd, 4th, and 10th day with the main following results (Table 1). Sample 4 was taken 10 days after childbirth. It was a transitional milk; therefore, some components were close to those of a mature milk and some were not yet. The mean macronutrient compositions of the milk are presented in Table 2. 

The Miris HMA^TM^ device is usually used to measure the energy, fat, carbohydrate, and protein content of human milk from mothers who have given birth prematurely or from human milk donors in order to assess the need to fortify their milk for preterm infants. The Miris HMA^TM^ device quantitatively measures the concentration of fat, carbohydrate, protein, total solids, and energy of human milk. The analytical technique used in Miris HMA^TM^ is a combination of established mid-infrared (mid-IR) transmission spectroscopy principles and a patented innovation. Miris HMA^TM^ is registered as a Medical Device in Europe, Japan, and the United States and applied in Human Milk Bank Gynecological and Obstetrics Hospital, Poznan University of Medical Sciences. 

According the cytological and microbiological diagnoses, literature review, and our own clinical experience, the diagnosis of rusty pipe syndrome was made. We encouraged our patient to continue breastfeeding. The neonatal weight gain was continuously controlled and within the normal range.

## 3. Discussion

The presence of an increased number of erythrocytes in prenatal milk and in colostrum is called rusty pipe syndrome. The estimated incidence rate of this syndrome is 0.1% in the population of breastfeeding women [5]. It affects primiparas, with bloody discharge observed in both breasts. The features of rusty pipe syndrome include an early onset (connected with the commencement of colostrum secretion), normal microbiological culture and cytological test results, the complete absence of blood-stained colostrum from the 5th day of life, the absence of any harmful effects for the mother and the baby, and no recurrence of the symptoms at later lactation stages and after subsequent childbirth [5,13]. The most likely reason for this coloring of the milk is the cracking of the walls of the thin blood vessels present in the breast stroma, which may take place during the proliferation and branching of milk ducts that occurs during lactogenesis I [10,11,12]. Merlob et al. [5] also suggested that individual predispositions to the increased permeability of capillaries, which in some women is manifested in easier bruising and more frequent gingival and nasal bleeding, may also underlie the condition, but this requires further research. 

Bloody discharge from the breast may also be caused by intraductal papilloma—a benign hyperplastic breast lesion. However, in such cases, the bleeding usually involves only one breast, persists for a longer period, and requires extended diagnosis [12,15]. A pre-pregnancy diagnosis of fibroadenoma is not a contraindication to breastfeeding, as it only requires regular check-ups and the supervision of an oncologist. Fibrocystic breast disease is the most common breast lesion, representing about 50% of benign breast diseases [16,17].

Our patient had low platelet counts both before and after childbirth. Thus far, there are no published data suggesting that low platelets can be a risk factor for rusty pipe syndrome. In our case, the low platelet counts could be one of the factors involved in this phenomenon. 

Medical indications for the use of a formula during a stay in the ward, according to the applicable recommendations of the Polish Neonatological Society, may occur because of the newborn or the mother [18,19]. This approach was followed in our unit. 

Due to the rarity of the occurrence of rusty pipe syndrome and its correlation with regurgitation and gastric residuals (green, brown, blood-stained discharge), there is a general fear of latching a newborn or continuing breastfeeding if the regurgitation persists. The mother may be afraid of feeding a neonate with bloody secretion; however, her milk is the most suitable food for a baby starting the first hours of its life [13,18,19,20,21,22,23]. Usually, mothers are not able to observe their colostrum color. Doubts may appear when the newborn’s regurgitated gastric content is brown or blood-stained. This may lead to the suspicion of intestinal bleeding and be the reason for further investigation. If the mother expresses the milk, it is easy to see the color of the discharge.

Recent methodologies have allowed us to analyze the composition of mothers’ milk almost at the bedside. In this case of rusty pipe syndrome, a composition analysis was performed. According to our literature review, no human milk analysis had previously been performed among mothers affected by rusty pipe syndrome. Our analysis revealed that blood-stained colostrum has a higher protein load and lower fat load in comparison to the mean macronutrient composition of milk [13,14] and a transient lower caloric load on the 3rd and 4th lactation day, although the mean macronutrient composition is variable and depends on many factors [13,14,15]. An exact maternal milk sample analysis may be crucial if a baby is born prematurely or extremely prematurely, but this was not the object of analysis in this case. We considered that our patient’s milk was appropriate for her baby because the newborn’s body mass stayed within a normal range.

Since breast milk has an indisputable invaluable impact on the newborn’s further development and there is no connection between rusty pipe syndrome, as described by the literature, and any pathologic processes, it is crucial to encourage mothers to maintain breastfeeding even if they observe blood-stained colostrum [6,20,21,22,23]. Special support should be provided from the moment of birth by medical, nursing, and obstetric staff and by lactation consultants. It is essential that all healthcare professionals present a uniform approach to rare and atypical phenomena, including rusty pipe syndrome.

## 4. Conclusions

(1). The analysis of blood-stained colostrum and transitional milk showed some shifts in particular macronutrients but neonatal weight gain in this case was proper and we did not find any contraindications for breastfeeding.
(2). The presence of blood in the colostrum is not a contraindication for breastfeeding, but special attention should be paid when it is given to premature babies (including noting the color of regurgitations and gastric residuals);
(3). There is need for support and counselling for all mothers, especially when they experience atypical milk color;
(4). There is an urgent need to perform a prospective cohort study in order to reassess the estimation of rusty pipe syndrome and the cause of the presence of erythrocytes in human milk.

## Figures and Tables

**Table 1:** The patient’s milk composition.

Analyzed Parameter	Sample 1 (2nd Day after Birth) COLOSTRUM	Sample 2 (3rd Day after Birth) TRANSITIONAL MILK	Sample 3 (4th Day after Birth) TRANSITIONAL MILK	Sample 4 (10th Day after Birth) MATURE/ TRANSITIONAL MILK
Total protein (g/100 mL)	5.9	4.3	2.9	1.7
Nutritional protein (g/100 mL)	4.8	3.5	2.4	1.4
Fat (g/100 mL)	1.3	1.1	1.1	2.9
Carbohydrates (g/100 mL)	4.5	4.7	6.3	7.1
Energy (kcal/100 mL)	56	48	49	63
Total solids TS (g/100 mL)	11.9	10.3	10.6	-

**Table 2:** The mean macronutrient compositions of the milk [13,14].

Particular Parameter	COLOSTRUM	TRANSITIONAL MILK	MATURE MILK
Total protein (g/100 mL)	0.2–7.5	1.5 [13]	0.2–1.3
Nutritional protein (g/100 mL)	Lack of data		
Fat (g/100 mL) [13]	2.6	3.7	3.2–3.6
Carbohydrates (g/100 mL) [13]	6.6	6.9	6.7–7.8
Energy (kcal/100 ml)	56 [13]	67 [13]	65.76 ± 7.92 [14]
Total solids TS (g/100 mL) [14]	11.86 ± 0.95	11.86 ± 0.95	11.86 ± 0.95
